# A Thin Film Flexible Supercapacitor Based on Oblique Angle Deposited Ni/NiO Nanowire Arrays

**DOI:** 10.3390/nano8060422

**Published:** 2018-06-11

**Authors:** Jing Ma, Wen Liu, Shuyuan Zhang, Zhe Ma, Peishuai Song, Fuhua Yang, Xiaodong Wang

**Affiliations:** 1 Engineering Research Center for Semiconductor Integrated Technology, Institute of Semiconductors, Chinese Academy of Science, Beijing 100083, China; majing@semi.ac.cn (J.M.); liuwen519@semi.ac.cn (W.L.); shuy@semi.ac.cn (S.Z.); mazhe@semi.ac.cn (Z.M.); pssong@semi.ac.cn (P.S.); fhyang@semi.ac.cn (F.Y.); 2 College of Materials Science and Opto-Electronic Technology, University of Chinese Academy of Sciences, Beijing 101408, China; 3 School of Electronic, Electronical and Communication Engineering, University of Chinese Academy of Sciences, Beijing 101408, China; 4 State Key Laboratory for Superlattices and Microstructures, Institute of Semiconductors, Chinese Academy of Sciences, Beijing 100083, China; 5 School of Microelectronics, University of Chinese Academy of Sciences, Beijing 101408, China

**Keywords:** flexible, NiO, oblique angle deposition, supercapacitor

## Abstract

With high power density, fast charging-discharging speed, and a long cycling life, supercapacitors are a kind of highly developed novel energy-storage device that has shown a growing performance and various unconventional shapes such as flexible, linear-type, stretchable, self-healing, etc. Here, we proposed a rational design of thin film, flexible micro-supercapacitors with in-plane interdigital electrodes, where the electrodes were fabricated using the oblique angle deposition technique to grow oblique Ni/NiO nanowire arrays directly on polyimide film. The obtained electrodes have a high specific surface area and good adhesion to the substrate compared with other in-plane micro-supercapacitors. Meanwhile, the as-fabricated micro-supercapacitors have good flexibility and satisfactory energy-storage performance, exhibiting a high specific capacity of 37.1 F/cm^3^, a high energy density of 5.14 mWh/cm^3^, a power density of up to 0.5 W/cm^3^, and good stability during charge-discharge cycles and repeated bending-recovery cycles, respectively. Our micro-supercapacitors can be used as ingenious energy storage devices for future portable and wearable electronic applications.

## 1. Introduction

With the rapid development of miniaturized electronic devices in recent years, there are great demands for flexible and micro energy storage devices at the same time. Flexible micro-supercapacitors (MSCs), as one of the most hopeful emerging energy storage devices, have shown great potential as power sources for portable and wearable electronic (PWE) products due to the advantages of high power density, fast charging capability, long life, high security, and varied changeable shapes [1,2,3,4,5,6,7,8,9,10]. So far, many types of flexible MSCs have been reported, such as in-plane supercapacitors, fiber supercapacitors, and branched supercapacitors. The in-plane interdigital MSC, which has interdigital shape electrodes with many dense micro fingers on a thin film substrate, was popular among many kinds of flexible MSCs. Compared with other types of MSCs, this type of flexible in-plane interdigital MSC can be prepared by traditional device processing technology to achieve a high specific surface area, small size, and high capacitance, and does not require complicated fabrication processes, multiple steps, or high cost. These superiorities render in-plane interdigital MSCs as powerful candidates for application in miniaturized portable electronic devices.

Due to above advantages, the in-plane interdigital MSC has been widely studied, and achieved remarkable results. In 2014, Xu et al. [11] reported a novel stack-integrated graphene photo-supercapacitor (PSC) thin-film device; they spread the graphite liquid coating on the surface of the substrate, which gained a capacitance of 896.77 uF/cm^3^ and a stack capacitance of 8.01 F/cm^3^ and a maximum energy density of 0.621 mWh/cm^3^ with a power density of 0.782 W/cm^3^. In 2015, Wu et al. [12] reported a flexible two-dimensional Ni(OH)_2_ nanoplate MSC, which achieved a specific capacitance of 8.80 F/cm^3^ at the scan rate of 100 mV/s, and the MSCs reached an energy density of 0.59 mWh/cm^3^ and a power density of up to 1.80 W/cm^3^. Although these in-plane interdigital MSCs exhibited a relatively high operating voltage and capacity, there are still some limitations. For example, the active materials on electrodes were easily stacked together, which led to the low specific surface area. In traditional processing of in-plane interdigital MSCs, most of the electrode materials were spin-coated on the substrate, which were easily peeled off when used in flexible applications. The performance of the device also needs further improvement to be suitable for real applications. Thus, some approaches could be proposed to improve the performance of in-plane interdigital MSCs to increase the energy-storage performance and enhance the robustness of the electrode materials, such as three-dimensional nanostructures designed to increase the specific surface area, enhance the adhesion between electrodes and the substrate, and optimize the size of the device. Oblique angle deposition [13,14,15] is a technique to form three-dimensional oblique nanowire arrays by electron beam evaporation, which allows the material to grow on a tilted substrate. It can be used to prepare high specific surface area, non-binder electrodes for interdigital MSCs, thereby increasing the capacity and energy density of MSCs and improving the flexibility by making the material more firm on the substrate. In 2016, Vasudevan Kannan et al. [16] demonstrated the electrochemical characteristics of NiO nanocolumnar electrode films prepared by oblique angle deposition technique for the first time, and they also proved that the supercapacitor showed its highest supercapacitance value when the tilt angle was 75°. However, they used copper sheets as the substrate, which may limit its application in portable and wearable electronic applications.

So in this work, oblique Ni/NiO nanowire arrays were grown directly on polyimide (PI) substrate through electron-beam evaporation with oblique angle deposition technology, to form interdigital electrodes for high performance flexible plane-interdigitated MSCs. The electrodes formed by this method have a high specific surface area and good adhesion to the substrate compared with other in-plane MSCs. Meanwhile, the as-fabricated MSCs show superior flexibility and outstanding energy-storage performance. This kind of plane-interdigitated MSC showed a high specific capacity of 37.1 F/cm^3^, a high energy density of 5.14 mWh/cm^3^, a power density up to 0.5 W/cm^3^, and good stability during repeated bending-recovery tests and charge-discharge cycles, respectively. The capacitance maintained nearly 94.7% of its initial value after 10,000 charge/discharge cycles. This flexible plane-interdigitated MSC can be used as ingenious energy storage devices for future portable and wearable electronic applications, with high energy-storage capacity and a long-lasting bending life.

## 2. Materials and Methods

### 2.1. NiO Nanowires-Based MSC Growth Process

PI films were cleaned by acetone, ethanol, and distilled water. Photolithography was then performed to get the interdigital pattern. Here, the width and spacing between electrodes were 200 µm. There were 12 electrodes on both sides, and electrodes were arranged uniformly. The width of the electrodes connecting the interdigital were 1000 μm in width and 1 cm in length. The effective area of the capacitor was 0.7465 cm^2^. A Ti (50 nm)/Au (20 nm) film was deposited on a PI substrate through sputtering, followed by a film of oblique angle deposited Ni nanowires (300 nm) by electron beam evaporation. The tilt angle of the substrate was 75°. Then, the sample was rinsed in acetone to remove redundant photoresist, followed by an annealing procedure of Ni nanowires at 300 °C for 2 h to get NiO shell on the nanowires. Finally, the polyvinyl (PVA)/potassium hydroxide (KOH) gel electrolyte was coated on the surface of interdigital electrodes in a thickness of about 2 µm, and then covered with a thin polydimethylsiloxane (PDMS) film. Thus, a high performance flexible plane-interdigitated MSC was completed.

The polyvinyl alcohol (PVA)/KOH polymer electrolyte was synthesized as follows: PVA (5 g) were dissolved in deionized (DI) water (45 mL) with stirring at 92 °C for 10 min. Then, KOH (5.6 g) were dissolved in DI water (5 mL). Finally, those two solutions above were mixed together with stirring at 60 °C to get clear solution [17].

### 2.2. Materials Characterization

The morphology and element analysis of as-prepared products were characterized by scanning electron microscopy (SEM, FEI NanoSEM650, Hillsboro, OR, USA), transmission electron microscopy (TEM; FEI Tecnai G2 F20, Hillsboro, OR, USA), X-ray photoelectron spectroscopy (XPS, Thermo escalab 250Xi, Waltham, MA, USA) and Raman spectroscopy (Renishaw inVia, New Mills, Gloucestershire, UK) with a 532-nm laser. Crystal structures were characterized with an X-ray diffractometer (XRD, Bruker D8 ADVANCE, Billerica, MA, USA) with radiation from a Cu-Kα radiation. Capacitance-voltage (CV) properties, galvanostatic charge/discharge measurements, electrochemical impedance spectroscopy (EIS), and the cycling performance of samples were recorded by a Chenhua CHI760E electrochemical workstation.

### 2.3. Calculation

The specific capacitance of the MSC was calculated by using the following equations [12,18,19]:

*C* = *I*·Δ*t*/Δ*V·S* (1)

Here, *C* represents the capacitance, *I* represents the current, Δ*t* represents the discharging time, Δ*V* is the applied potential, and *S* is the area of interdigital electrodes.

The energy density (*E*) and power density (*P*) of the device were calculated as follows:

*E* = *C*·Δ*V*^2^/7200·*V* (2)

*P* = *E*/Δ*t* (3)

where *V* is the volume of the as-packaged MSC.

## 3. Results

**Figure 1:**
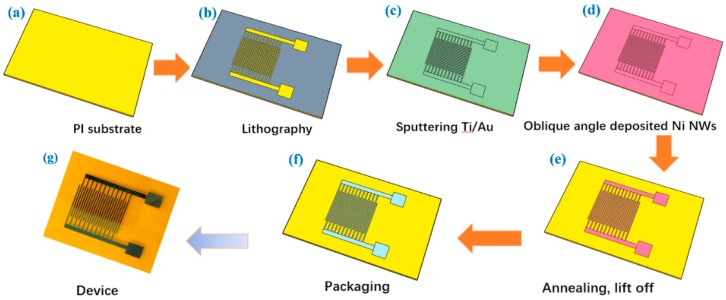
The schematic fabrication process of flexible micro-supercapacitors (MSCs). (**a**–**f**) Steps of preparing substrate, lithography, sputtering Ti/Au, oblique angle-depositing Ni nanowires annealing and packaging, respectively; (**g**) photograph of the as-fabricated flexible supercapacitor device.

Figure 1 demonstrates the typical fabrication procedures of the interdigital MSCs with the Ni/NiO nanowire arrays as electrodes. PI was used as the substrate on which photolithography was performed to get the desired pattern. The interdigital shape pattern was shown in Figure S1. Then, a Ti (50 nm)/Au (20 nm) film was sputtered on the PI substrate, followed by oblique angle deposited EDXNi nanowires film (300 nm), as shown in Figure 1c,d. The growth mechanism of Ni nanowires prepared by oblique angle deposition technology was shown in Figure S2. As the evaporant nucleates on the substrate, the region behind the nucleus does not receive any further vapor, because this region falls in the “shadow” of the nucleus. Therefore, vapor will only be deposited onto the nucleus, after which the columnar structure develops [20]. The shadowing effect introduces preferential growth on taller surface heights, and therefore enhances the island formation, even in the absence of surface diffusion, which does not exist at normal incidence [21]. The atoms excited by the electron beam are evenly deposited on the horizontal substrate, thus uniformly forming dense films. Moreover, the wavelength selection that gives rise to quasiperiodic morphologies has been proven to exist during oblique angle growth, which was not observed for continuous films deposited at normal incidence [21]. Researchers [20,21,22,23,24,25,26] have done a lot of work in this field, so according to the result, the tilt angle of the substrate of 75 ° and the evaporation rate of 0.15 nm/s were chosen to carry the electron beam evaporation, in order to get a uniform surface density of nanowire arrays [16]. After removing the redundant photoresist, the next step was annealing to oxidate the Ni nanowire arrays (Figure 1e). Finally, PVA/KOH gel electrolyte was coated on the surface of the sample, and the device was packaged with a thin PDMS film to get a high performance flexible supercapacitor (Figure 1f,g).

**Figure 2:**
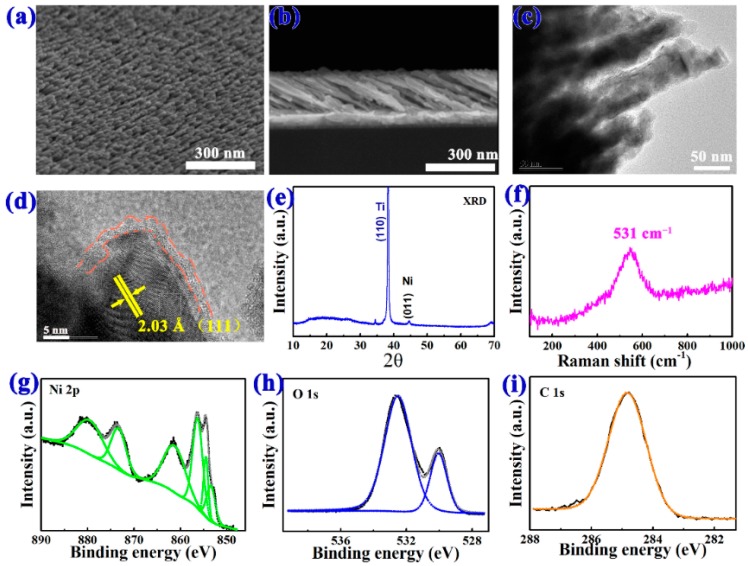
(**a**,**b**) Top-view and cross-section images of the nanostructured layer prepared on a polyimide (PI) substrate; (**c**) transmission electron microscopy (TEM) image of the oblique nanowire arrays; (**d**) high-resolution TEM (HRTEM) image of the nanowire; (**e**) X-ray diffractometer (XRD) patterns of the nanowires on the substrate; (**f**) Raman spectra measured on the surface of nanowires using a 532-nm laser; (**g**) Ni 2p; (**h**) O 1s and (**i**) C 1s X-ray photoelectron spectroscopy (XPS) spectra.

To further evaluate the form and detailed microstructures of the as-fabricated oblique nanowires, SEM and TEM were then employed, and the results are presented in Figure 2a–d. Figure 2a,b display the top-view and cross-section images of the nanostructured layer prepared on a PI substrate through oblique angle deposition technology. The pictures show that these layers presented a microstructure formed by tilted nanowires and a total thickness of 300 nm. The diameter of the NiO nanowires ranges between 30–50 nm, while the length of the nanowires is about 450 nm, and the inclination of the nanowires is about 40°. We can also calculate that the packing density of the NiO nanowires was 84.8% from the SEM images. This kind of tilted nanowires could enhance the specific surface area greatly. After the Brunauer-Emmett-Teller (BET) surface area measurement, we found that the BET surface area of the oblique NiO nanowires is as high as 658 m^2^/g. Figure 2c demonstrates the TEM image of the nanowires, which is in good agreement with the SEM results. Figure 2d demonstrates the high-resolution TEM (HRTEM) image of a nanowire. The clearly resolved lattice fringes in the core of the nanowire show that the d-spacing of 0.203 nm could be indexed to the (111) crystal planes of the Ni phase. A 3-nm thick oxide layer covered the Ni core, which was NiO formed in the annealing process. The distribution of the oxygen can be seen from the Energy Dispersive X-ray (EDX) mapping of the TEM image of the nanowires, as shown in Figure S3.

The element analysis of nanowires was further checked by using X-ray diffraction (XRD), Raman spectroscopy, and XPS. Figure 2e showed the XRD patterns of the annealed nanowires on a PI substrate. Peaks at 2θ = 38.48° were indexed to the (110) planes of the sputtered Ti (JCPDS card No. #44-1288) [27] on the PI, and the peaks at 2θ = 44.52 corresponded to the (011) diffraction planes, which indicated the rhombic hexahedron phase of Ni (JCPDS card no. #45-1027) [28] due to its incomplete oxidation [29,30]. The peaks of NiO were not obtained in this curve, due to the thickness of the oxide layer. The existence of NiO was demonstrated by the Raman spectrum of the surface of the sample (Figure 2f), which showed one broad peak corresponding to the one-phonon longitudinal optical mode of NiO (LO at 531 cm^−1^) [29,31]. X-ray photoelectron spectroscopy (XPS) was investigated to confirm the valence states and composition of Ni and O in the nanowires. Figure 2g–i demonstrate the Ni 2p, O 1s, and C 1s peaks, which were analyzed by using the software of XPS peak version 4.1 [32]. The peaks at 870–885 eV and 850–865eV correspond to Ni 2P_1/2_ and Ni 2P_3/2_ levels, respectively [33,34,35], and the peaks located at 879.56 eV and 873.33 eV represent Ni 2P_1/2_, while peaks at 861.28 eV, 856.28 eV, and 854.49 eV are characteristic peaks of Ni 2P_3/2_ (Figure 2g). Peaks of O 1s are located at 530.03 eV and 532.52 eV, which can be attributed to the binding energy in O–Ni and O-C, respectively [36]. In Figure 2i, the C 1s peak at 284.8 eV illustrates the C–C bond [37].

**Figure 3:**
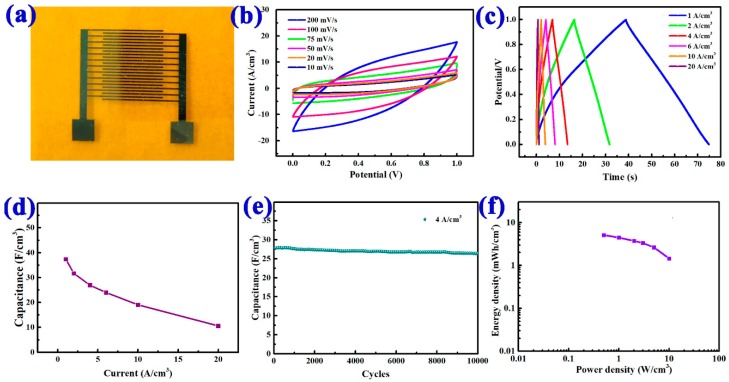
(**a**) Photos of the MSC devices; (**b**) capacitance-voltage (CV) curves at various scan rates; (**c**) Galvanostatic charge-discharge (GCD) at different currents measured in the voltage window of 0–1 V; (**d**) comparison of capacitances of the MSC devices at varied galvanostatic charge-discharge current densities; (**e**) capacitance retention on cycle number at a current of 4 A/cm^3^; (**f**) energy and powder densities of the MSC devices.

An as-fabricated plane-interdigitated MSC device was selected to carry out the electrochemical test with an electrochemical workstation to measure the electrochemical performance. Figure 3a shows the optical image of the as-prepared MSC device, and to further evaluate the performance of the MSC device, the electrochemical testing of the device was conducted, as displayed in Figure 3b–f. CV curves at the scan rate of 10–200 mV/s were depicted in Figure 3b. As the scan rate increased, closed areas of the CV curves became larger, while the quasi-rectangular shape indicated excellent capacitance [17,19,38]. For comparison, we also fabricated same-size MSCs with planar Ni film as the electrodes, and tested their CV curves (see Figure S4). It was found that the device with oblique NiO nanowires electrodes had better performance. Figure 3c presented the galvanostatic charging-discharging (GCD) curves of the device in the voltage window of 0–1 V. The GCD curves showed good symmetry, and the shape was close to a triangle, indicating an excellent capacitor performance. Figure 3d showed the relationship between specific capacitances and current densities. The specific capacitances evaluated from the CD curves were 37.1 F/cm^3^, 32.2 F/cm^3^, 27.1 F/cm^3^, 23.7 F/cm^3^, 19.2 F/cm^3^, and 10.6 F/cm^3^, corresponding to the current densities of 1 A/cm^3^, 2 A/cm^3^, 4 A/cm^3^, 6 A/cm^3^, 10 A/cm^3^, and 20 A/cm^3^, respectively. In addition, we chose another five identical MSCs made in the same batch as samples to measure the capacity performance; the result was shown in Figure S5. Due to the uniform nanowire arrays, the specific capacitances of these devices were very close, with a small error bar. Preeminent working stability under thousands of cycles is necessary for MSCs in real applications. This MSC exhibited excellent cycling stability at the current density of 4 A/cm^3^. As for the details, the capacitance was found to be 94.7% of its original capacitance (27.8 F/cm^3^) after the activation process of 10,000 cycles, as shown in Figure 3e, which indicated the excellent energy storage performance. Relations between energy density and power density of the MSC were displayed in Figure 3f. The device had a high energy density of 5.14 mWh/cm^3^ at a power density of 0.5 W/cm^3^. Even at a high power density of 10 W/cm^3^, the device still had an energy density of 1.46 mWh/cm^3^. The results are comparable to the plane-interdigitated MSCs that have been reported in recent literature, as displayed in Table S1.

**Figure 4:**
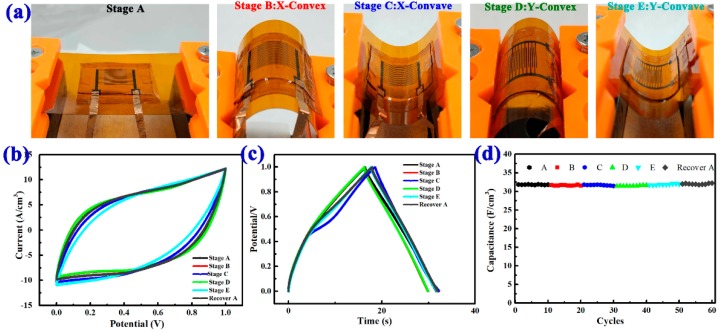
(**a**) Photos of a MSC at different bending states; (**b**) CV curves at 100 mV/s in straight and different bending states, respectively; (**c**) charge/discharge curves at a current of 2 A/cm^3^ in straight and different bending states, respectively; (**d**) capacitance performance under the different bending states.

The mechanical stability of the MSCs under bending states is a key parameter for practical use. So, here, we also studied the stability and flexibility of the MSC by bending the devices under different states. Figure 4a shows the schematic diagram of the MSCs in different bending states. Figure 4b demonstrates the CV curves of the MSCs under each stage. Meanwhile, the low performance degradation suggests the stable structural and electrochemical stability of the micro devices under bending. Figure 4c shows the charge/discharge curves at a current of 2 A/cm^3^ under the different angles, which demonstrate that our devices have excellent flexibility. Figure 4d presents the cycling performance of the MSCs at the current density of 2 A/cm^3^; we can find that the capacitance has no obvious degradation after every 10 cycles under each state, revealing its excellent cycling stability and excellent flexibility. Figure S6 shows the SEM images of the electrodes of MSCs after these flexibility tests. No change of the morphologies of the directly-grown NiO nanowires on the substrate was observed after bending. The length of nanowires was 450 nm, the thickness was 300 nm, the angle of the nanowires was 40 °, and bulk density was 85.1%, which is almost the same as the device before the bending test. This indicates that the surface morphology has no obvious difference from the initial morphology (Figure 2a,b), proving that the material can be firmly attached to the substrate. The electrode materials obtained by direct electron-beam evaporation have excellent adhesion to the substrate; they did not fall off after repetitive bending cycles. These results make it a good candidate for wearable device applications.

**Figure 5:**
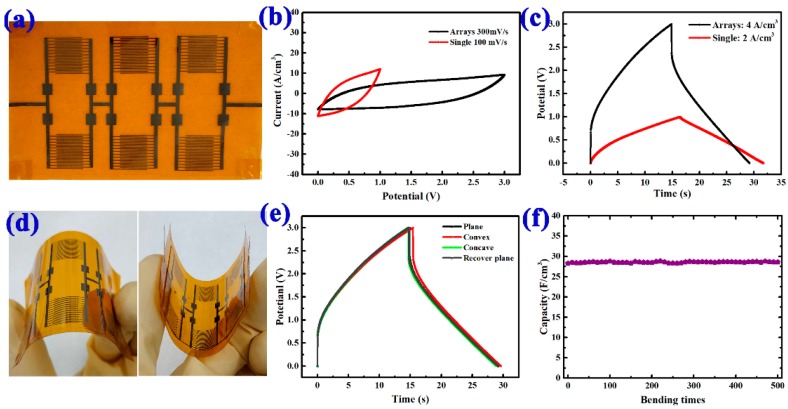
The integrated MSCs system based on six individual devices: (**a**) device position on the substrate; (**b**) CV curves of one MSC and an integrated arrays of six MSCs at scan rates of 100 mV/s and 300 mV/s, respectively; (**c**) galvanostatic CD curves of an array system of one MSC and six MSCs at the currents of 2 A/cm^3^ and 4 A/cm^3^, respectively; (**d**) photos of an integrated MSCs system; (**e**) flexibility performance of the integrated MSCs system based on six individual devices at different bending states; (**f**) the capacitance stability of the MSCs during repeated bending-recovery cycles at a galvanostatic current of 4 A/cm^3^.

Considering the practical application of the flexible MSCs, an integrated device system based on six individual devices was designed and fabricated, as shown in Figure 5a. The integrated MSCs system was connected in parallel with two groups of three in-series MSCs. Figure 5b compared the CV curves of a single MSC and the integrated arrays device system at scan rates of 100 mV/s and 300 mV/s, respectively. Figure 5c compared the galvanostatic CD curves of a single MSC and an integrated device system at currents of 2 A/cm^3^ and 4 A/cm^3^, respectively. These results indicated the superior potential of the MSCs to be patterned and integrated as a whole system to extend the capacity and working current for practical applications. The flexibility and stability of the MSC arrays on a PI substrate were then studied by bending the devices under different states, as demonstrated in Figure 5d,e. The capacitance exhibited no attenuation under different stages. Figure 5f showed cyclic stability; we found that the capacitance has no obvious degradation compared with its original value after 500 concave-restoring bending cycles, revealing its excellent cycling and mechanical stability. A good firmness of the electrode materials was also confirmed in this test.

## 4. Conclusions

In conclusion, a low-cost, facile, efficient, and versatile approach is developed for fabricating flexible plane-interdigitated MSCs. In-plane MSCs with versatile patterns based on interdigital, ultra-thin, and highly integrated electrodes are obtained by directly photolithography, and then successively depositing NiO nanowire arrays by an oblique angle deposition technique. As the specific surface area of oblique NiO nanowires is as high as 658 m^2^/g, the flexible plane-interdigitated MSCs show a high specific capacity of 37.1 F/cm^3^, a high energy density of 5.14 mWh/cm^3^, and a power density of up to 0.5 W/cm^3^, which is better than or comparable with recently reported results. The as-prepared MSCs also demonstrates excellent stability during 10,000 charge-discharge cycles. which can be attributed to the excellent flexibility of the ultra-thin integrated electrode and a strong binding force between the electrode materials and the substrate. Multiple MSCs can also be connected in series and parallel without additional interconnection circuits, and arbitrary shaped MSCs can be fabricated readily through modifying photolithography patterns. In addition, the as-prepared MSCs can be transferred to different substrates, which will enrich the design flexibility and fulfill fashion demands for the design of PWEs, and extensively extend application scenarios of MSCs in PWEs. The hand-drawing assisted method demonstrated in this work provides a low-cost, facile, efficient, and versatile approach for fabricating high-performance, flexible, shape customizable and compatible MSCs, and also paves a promising way for fabricating other wearable electronics.

## Supplementary Materials

The following are available online at http://www.mdpi.com/2079-4991/8/6/422/s1, Figure S1: the interdigital shape pattern of the in-plane interdigital MSCs; Figure S2. The growth mechanism of Ni nanowires prepared by oblique angle deposition technology; Figure S3: The EDX Mapping of the nanowires from TEM image; Figure S4: CV curves of NiO electrodes deposited at normal and oblique deposition angles 75° at a 100 mV/s scan rate; Figure S5: The capacitances of five identical MSC devices made in the same batch at varied galvanostatic charge-discharge current densities, with error bar; Figure S6: SEM images of the electrodes of MSCs after bending test; Table S1: Comparation of the plane-interdigitated supercapacitors reported in recent years.
