# Supplementary material for: A Thin Film Flexible Supercapacitor Based on Oblique Angle Deposited Ni/NiO Nanowire Arrays
Source: Nanomaterials (Basel). 2018 Jun 11;8(6):422. doi: 10.3390/nano8060422 (PMC6027448; doi:10.3390/nano8060422)
Supplement: Supplementary file 1 [file nanomaterials-08-00422-s001.pdf]

Supplementary Information

A Thin Film Flexible Supercapacitor Based on Oblique Angle Deposited Ni/NiO Nanowire Arrays

Jing Ma [1,2], Wen Liu [1], Shuyuan Zhang [1,2], Zhe Ma [1,2], Peishuai Song [1,3], Fuhua Yang [1,4] and Xiaodong Wang [1,5,*]

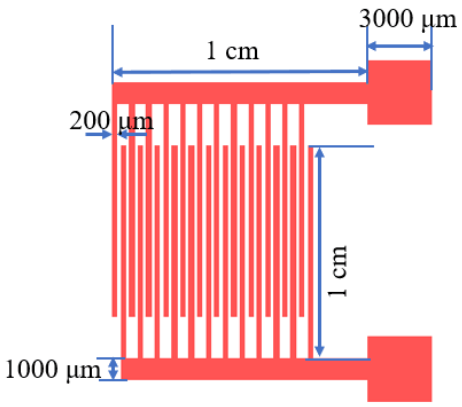

Figure S1. The interdigital shape pattern of the in-plane interdigital MSCs.

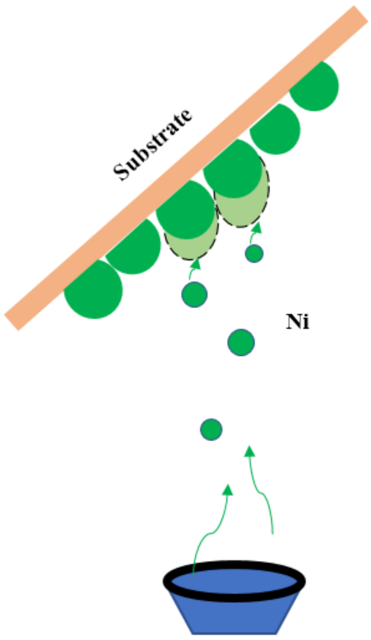

Figure S2. The growth mechanism of Ni nanowires prepared by oblique angle deposition technology.

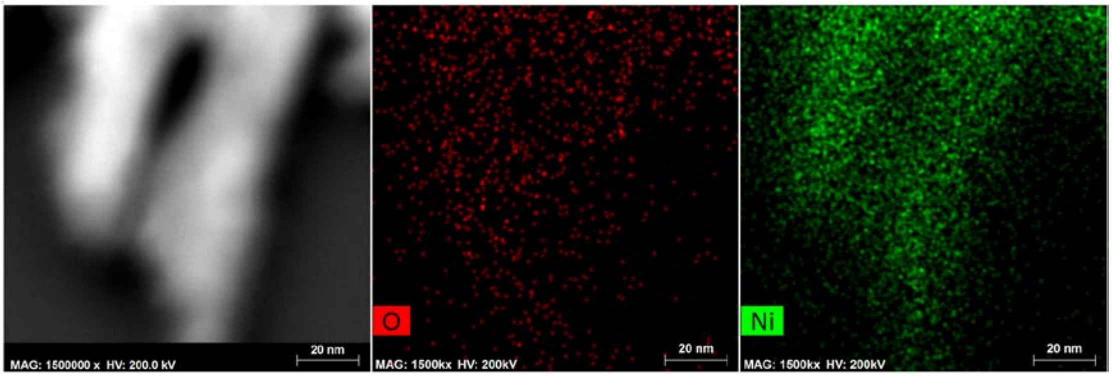

Figure S3. The EDX Mapping of the nanowires from TEM image.

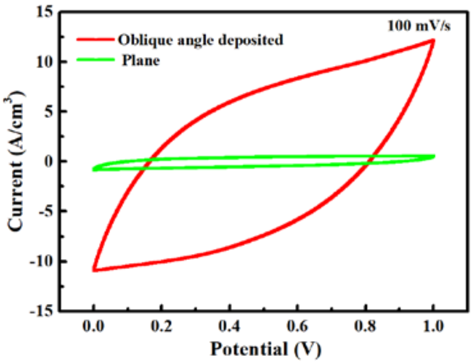

Figure S4. CV curves of NiO electrodes deposited at normal and oblique deposition angles 75° at a 100 mV/s scan rate.

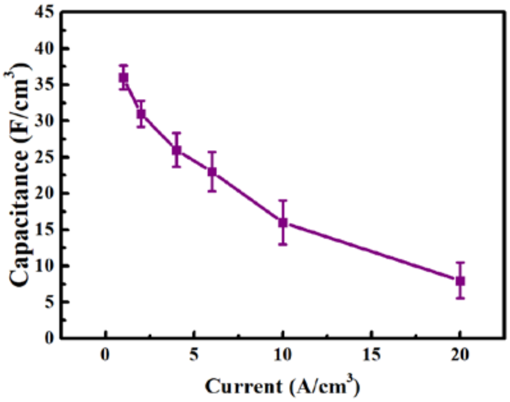

Figure S5. The capacitances of five identical MSC devices made in the same batch at varied galvanostatic charge-discharge current densities, with error bar.

Table S1. Comparation of the plane-interdigitated supercapacitors reported in recent years.

Ref.	Materials	Capacity	Cycles	Energy density	Power density	Flexible
[1]	$Ni(OH)_2$ nanoplates	8.80 F/cm^3	10,000	0.59 mWh/cm^3	1.80 W/cm^3	Y
[2]	rGO–CNT composites	6.1 mF/cm^2 3.1 F/cm^3	1000	0.68 mWh/cm^3	77 W/cm^3	N
[3]	graphene	80.7 mF/cm^2 17.9 F/cm^3.	100,000	2.5 mWh/cm^3	495 W/cm^3	Y
[4]	MXene-based micro-supercapacitors	23 mF/cm^2	10,000	2.8 mWh/cm^3 2.3 mWh/cm^3	225 mW/cm^3 744 mW/cm^3	Y
[5]	graphene/ polymer (PE) composite	95 mF/cm^2	10,000	8.4◎ ×◎ 10^{-3} mWh/cm^{-2}	2.9 mW/cm^2	N
[5]	AC/PE composite	134 mF/cm^2	10,000	12◎ ×◎ 10^{-3} mWh/cm^{-2}	1.3 mW/cm^2	N
[6]	MnO_2 nanoparticle	338.1 F/g	500			N
[7]	onion-like carbon (OLC) electrodes	1.3 F/cm^3	10,000		1 kW/cm^3	N
Our work	Ni/NiO NWs	37.1 F/cm^3	10,000	1.86 Wh/cm^3	180 W/cm^3	Y

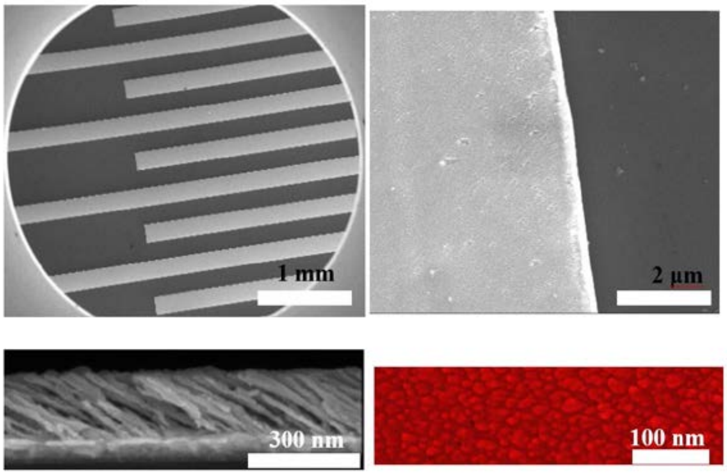

FigureS6. SEM images of the electrodes of MSCs after bending test.

Reference

1. Wu, H.; Jiang, K.; Gu, S.; Yang, H.; Lou, Z.; Chen, D.; Shen, G. Two-dimensional $Ni(OH)_2$ nanoplates for flexible on-chip microsupercapacitors. Nano Research 2015, 8, 3544–3552.
2. Beidaghi, M.; Wang, C. Micro-supercapacitors based on interdigital electrodes of reduced graphene oxide and carbon nanotube composites with ultrahigh power handling performance. Advanced Functional Materials 2012, 22, 4501–4510.
3. Wu, Z.S.; Parvez, K.; Feng, X.; Mullen, K. Graphene-based in-plane micro-supercapacitors with high power and energy densities. Nat Commun 2013, 4, 2487.
4. Jiang, Q.; Wu, C.; Wang, Z.; Wang, A.C.; He, J.-H.; Wang, Z.L.; Alshareef, H.N. Mxene electrochemical microsupercapacitor integrated with triboelectric nanogenerator as a wearable self-charging power unit. Nano Energy 2018, 45, 266–272.

5. Pu, J.; Wang, X.; Zhang, T.; Li, S.; Liu, J.; Komvopoulos, K. High-energy-density, all-solid-state microsupercapacitors with three-dimensional interdigital electrodes of carbon/polymer electrolyte composite. Nanotechnology 2016, 27, 045701.
6. Xue, M.; Xie, Z.; Zhang, L.; Ma, X.; Wu, X.; Guo, Y.; Song, W.; Li, Z.; Cao, T. Microfluidic etching for fabrication of flexible and all-solid-state micro supercapacitor based on MnO_2 nanoparticles. Nanoscale 2011, 3, 2703–2708.
7. Pech, D.; Brunet, M.; Durou, H.; Huang, P.; Mochalin, V.; Gogotsi, Y.; Taberna, P.L.; Simon, P. Ultrahigh-power micrometre-sized supercapacitors based on onion-like carbon. Nat Nanotechnol 2010, 5, 651–654.
